# Supplementary material for: Content-rich biological network constructed by mining PubMed abstracts
Source: BMC Bioinformatics. 2004 Oct 8;5:147. doi: 10.1186/1471-2105-5-147 (PMC528731; doi:10.1186/1471-2105-5-147)
Supplement: Additional File 2 — The original results of the above study (non-essential files are deleted to keep the file size under the limit set by BMC bioinformatics). [file 1471-2105-5-147-S2.bz2 › chilibotAdditionalFile2/dip05/240ID9063449E787/html/MMP1_TIMP1.html]

 


 **MMP1** and **TIMP1** 
  
Found 2107 abstracts in PubMed, retrieved 05.  
 

 What does Google say? 
 PDF only 
| .edu only 

---

**Interactive relationship** (e.g. stimulation, inhibition, etc)

**Inhibitory relationship**- The minidegradome constituted of two matrix metalloproteinases MMP  [ **MMP1** ] , i.e. MMP  [ **MMP1** ]  2 and MT1 MMP  [ **MMP1** ] , associated with tissue inhibitor of metalloprotease 2 TIMP  [ **TIMP1** ]  2 and integrin alpha v beta 3 or CD 44, is mainly involved in such invasive program.  Ref: 12888258 Biomed Pharmacother, 2003
**Neutral relationship**

**Non-interactive relationship** (e.g. studied together, co-existance, homology, etc.)

- Our findings establish an unexpected, MMP  [ **MMP1** ]  independent mechanism for TIMP  [ **TIMP1** ]  2 inhibition of endothelial cell proliferation in vitro and reveal an important component of the antiangiogenic effect of TIMP2 in vivo.  Ref: 12887919 Cell, 2003
- OBJECTIVES The present study was carried out to clarify the role of matrix metalloproteinase 2 and 9 MMP  [ **MMP1** ]  2 and MMP  [ **MMP1** ]  9, tissue inhibitor of metalloproteinase 1  [ **TIMP1** ]  TIMP 1  [ **TIMP1** ]  and the extent of neovascularization in the clinicopathologic behavior of non Hodgkin s lymphomas.  Ref: 12890147 Eur J Haematol, 2003
- We demonstrate here that TIMP  [ **TIMP1** ]  2 abrogates angiogenic factor induced endothelial cell proliferation in vitro and angiogenesis in vivo independent of MMP  [ **MMP1** ]  inhibition.  Ref: 12887919 Cell, 2003
- However, the mRNAs coding for urokinase type PA uPA, membrane type 1 MMP  [ **MMP1** ]  MT1 MMP  [ **MMP1** ] , and tissue inhibitor of metalloproteinases  [ **TIMP1** ]  type 3 TIMP  [ **TIMP1** ]  3 were constantly expressed in the mouse CL throughout its whole life span.  Ref: 12890575 Mol Cell Endocrinol, 2003
- Among the patients with strong grades 2 and 3 MMP  [ **MMP1** ]  9 staining positivity for TIMP 1  [ **TIMP1** ]  indicated a trend toward a more favorable prognosis.  Ref: 12890147 Eur J Haematol, 2003
- METHODS Paraffin embedded histologic sections from 57 patients with aggressive non Hodgkin s lymphomas were stained with MMP  [ **MMP1** ]  2, MMP  [ **MMP1** ]  9, TIMP 1  [ **TIMP1** ] , and factor VIII antibodies to correlate the expression of these markers to the clinical disease characteristics.  Ref: 12890147 Eur J Haematol, 2003
- 0.001, and the MMP  [ **MMP1** ]  inhibitor TIMP  [ **TIMP1** ]  2 P = 0.025.  Ref: 12893184 Gynecol Oncol, 2003
- Gelatinases MMP  [ **MMP1** ]  2 and MMP  [ **MMP1** ]  9, TIMP 1  [ **TIMP1** ]  expression and the extent of neovascularization in aggressive non Hodgkin s lymphomas.  Ref: 12890147 Eur J Haematol, 2003
